# Insulitis and lymphoid structures in the islets of Langerhans of a 66-year-old patient with long-standing type 1 diabetes

**DOI:** 10.1007/s00428-020-02915-4

**Published:** 2020-08-24

**Authors:** Silke Smeets, Willem Staels, Geert Stangé, Pieter Gillard, Nico De Leu, Peter in’t Veld

**Affiliations:** 1 Diabetes Research Center, Vrije Universiteit Brussel (VUB), Brussels, Belgium; 2 Beta Cell Neogenesis (BENE), Vrije Universiteit Brussel (VUB), Brussels, Belgium; 3 Institut Cochin, INSERM, U1016, CNRS, UMR8104, Université de Paris, 75014 Paris, France; 4 Department of Pediatrics, Division of Pediatric Endocrinology, University Hospital of Brussels, Jette, Belgium; 5 Department of Endocrinology, University Hospital Leuven, Leuven, Belgium; 6 Department of Endocrinology, UZ Brussel, Brussels, Belgium

**Keywords:** Human, Type 1 diabetes, Insulitis, Pancreas, Tertiary lymphoid structure

## Abstract

Insulitis is a characteristic inflammatory lesion consisting of immune cell infiltrates around and within the pancreatic islets of patients with recent-onset type 1 diabetes (T1D). The infiltration is typically mild, both in terms of the number of infiltrating cells and the number of islets affected. Here, we present an unusual histopathological case study of a 66-year-old female patient with long-standing T1D, insulitis, and islet-associated lymphoid tissue. Most islets in the head of the pancreas of this patient were insulin-deficient, whereas the islets in the tail appeared normal. Insulitis was present in 0.84% of the insulin-containing islets and three islets had large lymphocytic infiltrates resembling tertiary lymphoid structures (TLS). Of note, this is the first description of potential TLS in the endocrine pancreas of a patient with T1D. Their association with a marked residual beta cell mass is of interest and may hint at new insights into disease progression and regulation of autoimmunity.

**Electronic supplementary material:**

The online version of this article (10.1007/s00428-020-02915-4) contains supplementary material, which is available to authorized users.

## Introduction

Type 1 diabetes (T1D) is considered to be caused by an autoimmune T cell–mediated destruction of insulin-producing beta cells in the pancreas leading to loss of glucose homeostasis [1]. At disease onset, most pancreatic islets have lost their normal beta cell complement but some still contain insulin. Islets with residual beta cells may show insulitis, a characteristic leucocytic infiltration rich in CD8+ T cells thought to mediate beta cell–specific autoimmunity [2, 3]. Mechanisms of autoimmune beta cell destruction have mainly been studied in animal models, especially that of the non-obese diabetic (NOD) mouse since the disease course in the latter shows many similarities with human T1D. Several important differences, however, exist between the NOD mouse model and patients, including the frequent presence of islet-associated tertiary lymphoid structures (TLS) in the mouse model. TLS are ectopic lymphoid organs that develop at sites of chronic inflammation in non-lymphoid tissues and resemble secondary lymphoid organs with neighboring B and T lymphocyte compartments. TLS are thought to be important for promoting antigen-specific humoral responses during chronic inflammation in several human autoimmune conditions but have not yet been reported in patients with T1D [2]. In NOD mice, TLS have been found to promote autoimmunity and chronic inflammation during diabetes progression [4, 5]. This is the first report of structures resembling TLS in the islets of Langerhans of a patient with T1D.

## Methods

### Case history

A 66-year-old Caucasian patient with T1D for 18 years died from intracerebral bleeding (case number DBB3450, Diabetes Biobank Brussels). She used multiple daily insulin injections, with a daily total dose of 12.5 U insulin aspart (NovoRapid®) and 8 U insulin detemir (Levemir®). The patient was positive for autoantibodies against GAD (41 U/ml; normal range < 1 U/ml) and had a susceptibility HLA-DQ genotype (DQA1*0301-DQB1*0301/ DQA1*0301-DQB1*0302). Her comorbidities were vitiligo, autoimmune gastritis, and neuropathy. Available clinical data are shown in Table S1. The pancreatic organ (63.5 g) was made available for organ donation and stored in a registered Biobank (Diabetes Biobank Brussels, approval number local ethics committee B.U.N. 143201941720) as formalin-fixed and paraffin-embedded or flash-frozen samples.

### Immunohistochemical analysis and morphometry

Sections were processed and stained for insulin, glucagon, synaptophysin, Ki67, CD3, CD4, CD8, CD20, CD21, CD23, CD31, CD45, CD68, and MECA-79 using immunofluorescence and immunoperoxidase staining (Supplementary Methods). All antibodies were validated in tonsil and pancreatic lymph node. Triple immunofluorescent stained sections for insulin, glucagon, and CD45 were digitally imaged using a Pathway 435 inverted fluorescence microscope (Becton Dickinson, San Jose, CA, USA) to obtain whole slide images. The number of insulin-containing islets, insulin-deficient islets, and islets showing CD45-positive insulitis was counted manually in 10–15 sections per pancreatic region (5 sections/tissue block—160 μm between each section). Islets of Langerhans were defined as structures with at least 10 insulin- and/or glucagon-positive cells. Insulitis was defined by the presence of ≥ 15 cells positive for the leucocyte common antigen CD45 in or immediately adjacent to an islet [3]. Digital images were analyzed by morphometry (IPLab; Becton Dickinson) to measure insulin-positive, glucagon-positive, and total pancreatic (DAPI-positive) areas. Relative beta and alpha cell areas were calculated by dividing the insulin- and glucagon-positive area by the total pancreatic area. The composition of the insulitic lesion was analyzed on slides triple stained for insulin and CD3 in combination with CD4, CD8, CD20, or CD68 and was expressed as the average amount of cells per islet ± SD. Islet cell replication was assessed by double staining for insulin and the replication marker Ki67 in 1000 nucleated insulin-positive cells and expressed as percent Ki67+ insulin+ cells. Every 40th sections of five (4 paraffin and 1 frozen) tissue blocks from the pancreas tail were stained for CD45 and synaptophysin and screened for TLS-like structures. Consecutive sections of islets with TLS-like structures were analyzed for the presence of insulin, CD3+ T lymphocytes, CD20+ B lymphocytes, MECA-79+ high-endothelial venules, and CD21+ or CD23+ follicular dendritic cells.

## Results

**Fig. 1:**
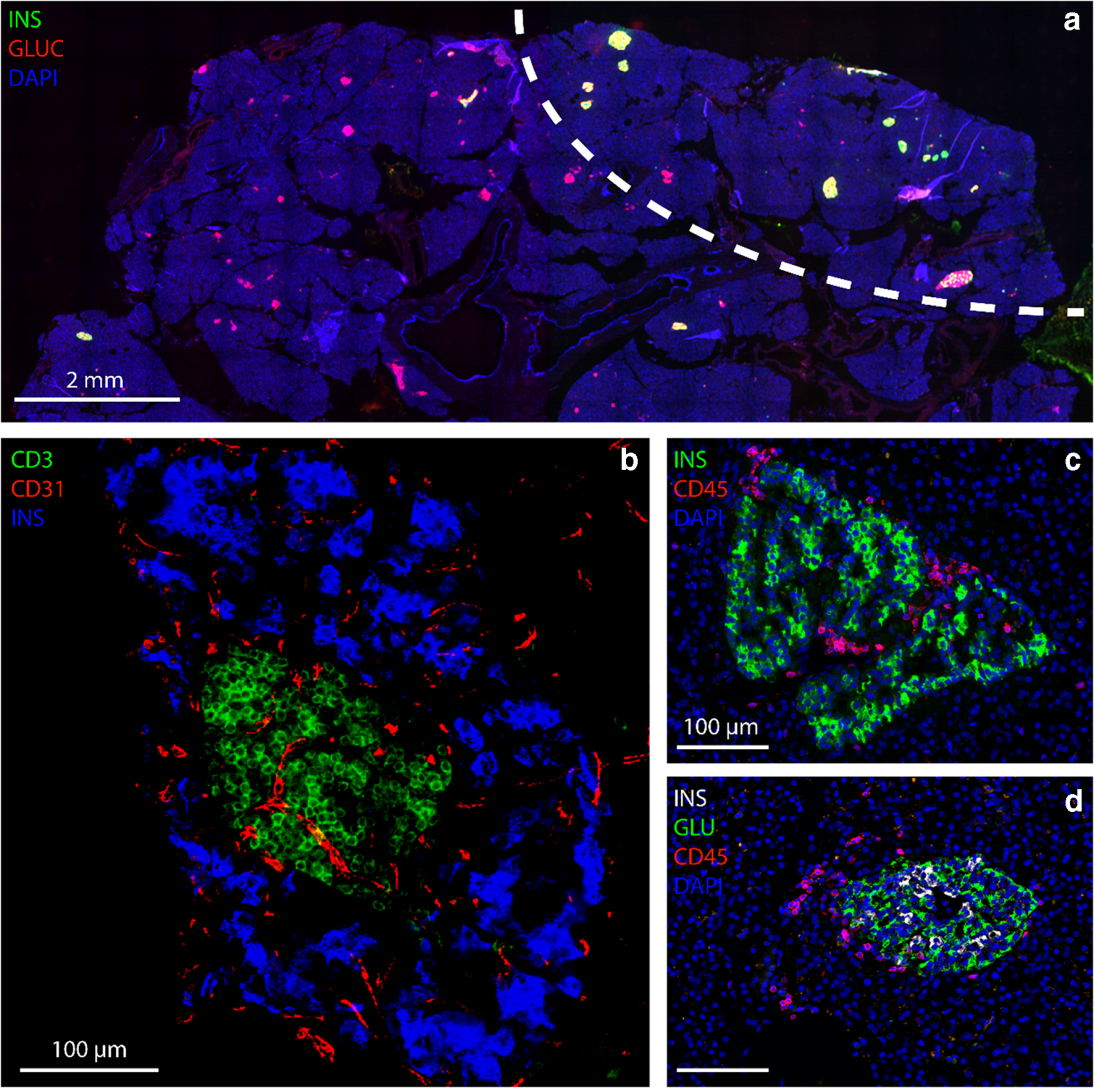
**a** Low magnification of a pancreatic section from the pancreas tail stained for insulin (green), glucagon (red), and DAPI (blue), showing a lobular distribution of insulin-containing islets (above the dotted line). **b** Section stained for insulin (blue), CD3 (green), and CD31 (red), showing a large intra-islet lymphocytic infiltration. **c** Section stained for insulin (green) and CD45 (red) showing an insulitic lesion. **d** Section stained for insulin (white), glucagon (green), and CD45 (red) showing a second insulitic lesion

Immunohistochemical staining for insulin, glucagon, and CD45 showed pseudo-atrophic insulin-deficient islets throughout the pancreas, with insulin-containing islets being virtually absent in the head region. In contrast, well-granulated insulin-containing islets, a near-normal relative beta cell area, and a markedly increased relative alpha cell area were found in the tail of the gland (Table 1), with insulin-containing islets showing a lobular distribution (Fig. 1a). Insulitis was present in a small fraction (0.84%) of the insulin-containing islets but was absent from all insulin-deficient islets. The insulitic infiltrate consisted predominantly of lymphocytes, especially CD8+ T cells (28.0 ± 14.2 cells/islet; mean ± SD) and to a lesser extent of CD4+ T cells (13.5 ± 19.5) and CD20+ B cells (5.7 ± 8.8). CD68+ macrophages were also observed (11.0 ± 6.8) (Fig. 1b, c). Beta cell replication in both insulitic and non-insulitic islets was < 0.1% and no Ki67+ lymphocytes were seen in the insulitic lesions.

**Table 1:** Quantification of beta cell area, alpha cell area, and number of insulin-containing and insulin-deficient islets in different pancreatic regions

	Beta cell area/total pancreatic area ± SD (%)	Alpha cell area/total pancreatic area ± SD (%)	Beta cell area/islet area ± SD (%)	Alpha cell area/islet area ± SD (%)	ICI	IDI
Ventral head	0.00 ± 0.01	0.65 ± 0.36	0.59 ± 0.68	99.41 ± 0.68	3	261
Dorsal head	0.00 ± 0.00	0.65 ± 0.47	0.22 ± 0.32	99.78 ± 0.32	1	939
Body	0.04 ± 0.06	0.88 ± 0.38	3.46 ± 4.10	96.54 ± 4.10	104	1371
Tail	0.49 ± 0.40	2.37 ± 0.79	14.23 ± 8.43	85.77 ± 8.43	488	1246

Data are expressed as mean relative beta/alpha cell area ± SD in relation to the total pancreatic area or as the mean relative beta/alpha cell area ± SD in relation to the total islet area (beta + alpha cell area) in 10–15 sections per pancreatic region. Data on islet subtypes (insulin-containing islets [ICI] and insulin-deficient islets [IDI]) are expressed as islet subtype counts in 10–15 slides

**Fig. 2:**
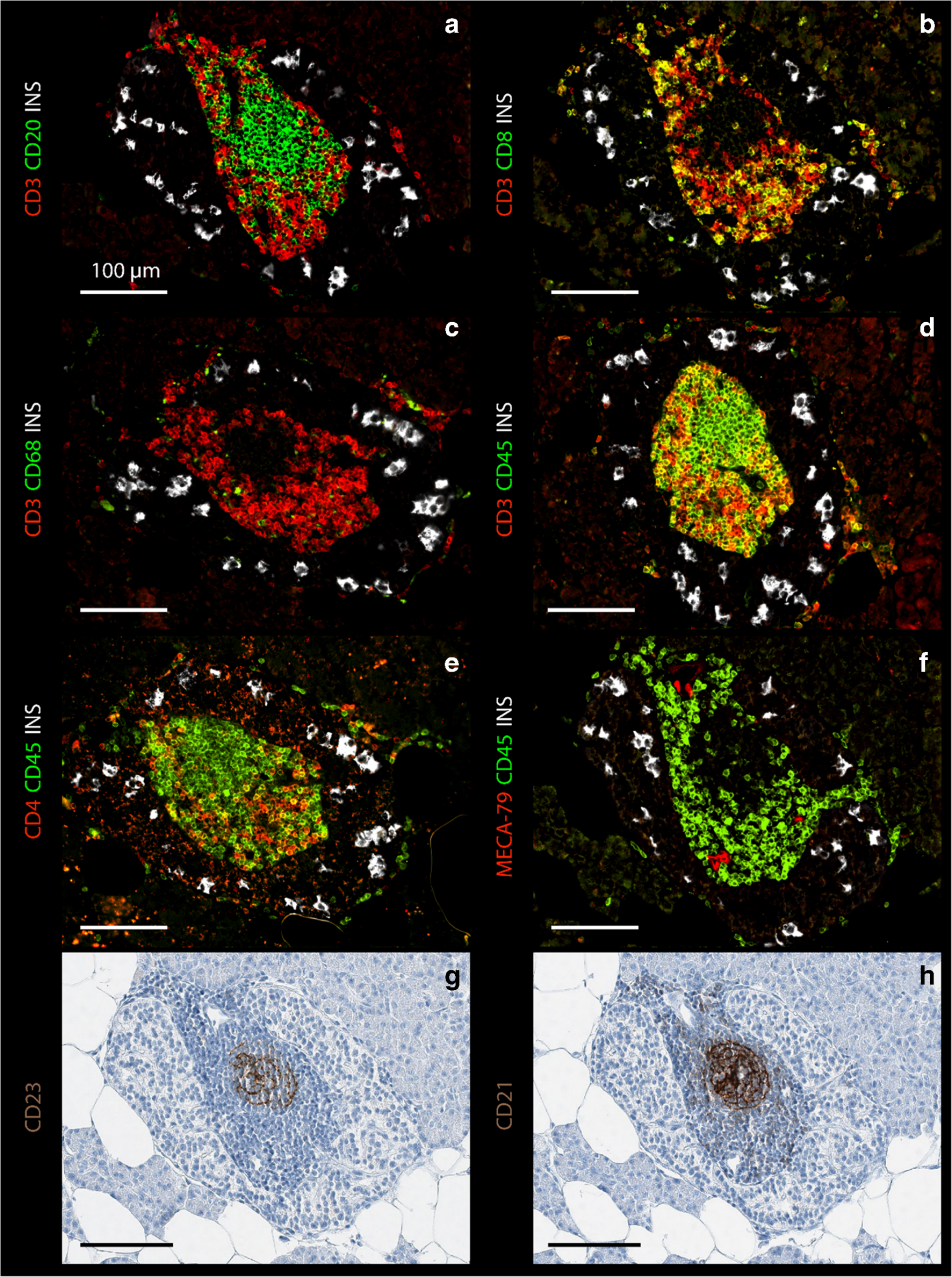
Consecutive sections from a TLS-like structure stained for **a**–**f** different combinations of insulin, CD3, CD20, CD8, CD68, CD45, CD4, and MECA-79, for **g** CD23, and **h** CD21 showing a large lymphocytic infiltrate with a medulla rich in CD20 B lymphocytes, CD23-, and CD21-positive follicular dendritic cells and a peripheral cortex of CD3, CD4, and CD8 T lymphocytes showing very few CD68-positive macrophages and the presence of MECA-79-positive high-endothelial venules

TLS-like structures were observed in three insulin-containing islets in the tail region of the gland (Fig. 1b, Fig. 2, and Figure S1). They were characterized by segregated T and B lymphocyte zones, MECA-79-positive high endothelial venules in the T lymphocyte zone, and CD21/CD23 follicular dendritic cells in the B lymphocyte zone (Fig. 2 and Figure S1). No TLS-like structures were observed outside the pancreatic islets or in other gland regions.

## Discussion

This case report describes the presence of insulitis and TLS-like structures in a 66-year-old female patient with long-standing T1D. TLS are ectopic aggregations of lymphoid cells formed under the influence of antigens during chronic inflammation. They have been described in chronic graft rejection and in different neoplastic, infectious, and autoimmune diseases in patients, in particular in organ-specific autoimmune disorders. TLS are characterized by a combination of five criteria: adjacent T and B lymphocyte zones, the presence of high endothelial venules, fibroblastic reticulum cells in the T cell zone, and presence of the AID enzyme and follicular dendritic cells [5]. In the present case study, three out of five of these criteria were met; for the two remaining criteria, the immunocytochemical data were equivocal. The lymphoid structures found in this patient were therefore named TLS-like. In animal models of T1D, in particular, the NOD mouse model, lymphoid structures in insulitic lesions are frequently seen [6]; these structures were shown to have the characteristics of fully functional TLS, capable of supporting in situ autoreactive B lymphocyte differentiation and are suggested to be critical for promoting autoimmunity and chronic inflammation [7]. The absence of TLS in patients with T1D has previously been used as an argument for a different etiopathogenesis of the human disease when compared with the rodent model [2]. The present case shows that lymphoid structures with structural characteristics of TLS, in particular adjacent T and B lymphocyte zones, the presence of high endothelial venules in the T cell area, and the presence of follicular dendritic cells, can occur in patients with T1D, albeit at low frequency.

In addition to TLS-like structures, the patient also showed insulitis, which is a rare finding in patients with long-standing T1D. Lymphocytic infiltrates are most frequently seen in the islets of patients with recent-onset disease [2, 8], and only two cases with moderate insulitis with an age of onset over 40 years and a long-standing (> 1 year) T1D have previously been reported [9, 10]. The patient was also characterized by a substantial residual beta cell mass in combination with alpha cell hyperplasia, with a substantially higher relative alpha cell area in the tail of the gland compared with the other pancreatic regions, and to values reported in non-diabetic controls [11]. Residual beta cells are not uncommon in patients with long-standing T1D, with approximately 50% of patients having low numbers of residual beta cells, often in a focal or lobular distribution pattern [8, 12, 13]. However, the present case shows exceptionally high numbers of residual beta cells, with a relative beta cell area in the tail part of the gland that is close to the values found in non-diabetic controls [14].

In a now classical model by Eisenbarth [15], it was proposed that the presence of autoantibodies indicates ongoing beta cell–specific autoimmune destruction and that the beta cell mass declines linearly with an increasing disease duration. More recently, this model has been challenged in view of the complexity of the disease and the heterogeneity between patients; the functional beta cell mass is now seen as a dynamic parameter, possibly reflecting episodes of beta cell regeneration and destruction [1]. In this context, the observation of a marked residual beta cell mass in the present case study in combination with TLS-like structures may point to the interesting possibility that such lymphoid structures do not necessarily reflect a process of chronic autoimmunity, but alternatively reflect a process of local tolerance mediated via regulatory T cells, facilitating beta cell survival. A more detailed analysis of these TLS-like structures as to their potential regulatory role would be of interest, as would a systematic search for TLS in T1D patients with evidence of residual beta cell function.

## Electronic supplementary material

ESM 1 (DOCX 801 kb)

## Data Availability

The detailed datasets generated during and/or analyzed during the current study are available from the corresponding author upon request.

## Abbreviations

T1D: Type 1 diabetes
TLS: Tertiary lymphoid structures
