# Supplementary material for: Insulitis and lymphoid structures in the islets of Langerhans of a 66-year-old patient with long-standing type 1 diabetes
Source: Virchows Arch. 2020 Aug 24;478(6):1209–14. doi: 10.1007/s00428-020-02915-4 (PMC8203531; doi:10.1007/s00428-020-02915-4)
Supplement: Supplementary file 1 — (DOCX 801 kb) [file 428_2020_2915_MOESM1_ESM.docx]

**Supplementary Material**

**Supplementary methods:**

**Orientation of the pancreas.** The ventral pancreas was defined by anatomy at the time of resection of the different tissue blocks and has been confirmed afterward by pancreatic polypeptide immunohistochemistry.

**Immunohistochemistry.** 4 µm sections of the paraffin-embedded tissue blocks and/or cryoblocks were immunohistochemically stained as followed: (1) To quantify the insulin-containing islets, insulin-deficient islets, the number of islets with insulitis and the relative beta and alpha cell area, we used triple immunofluorescent staining for guinea-pig anti-insulin (1:4000; DRC; VUB; Brussels; Belgium), rabbit anti-glucagon (1:2000; DRC) and mouse anti-CD45 (1:50; clone 2B11+PD7/26; Agilent Technologies; Heverlee; Belgium). This staining was performed on at least 10 paraffin sections (5 sections/tissue block) of each part of the pancreas with a distance of 160 µm between each section. (2) To characterize the composition of the insulitic lesions, consecutive paraffin sections of the sections with insulitis were triple stained for guinea pig anti-insulin and rabbit anti-CD3 (1:25; Agilent Technologies) in combination with mouse anti-CD68 (1:50; clone KP1; Agilent Technologies), mouse anti-CD8 (1:10; clone 1A5; Novocastra Reagents; Leica Microsystems; Wetzlar; Germany), mouse anti-CD20 (1:400; clone L26; Agilent Technologies), or rabbit anti-CD4 (ready-to-use; clone SP35; Ventana; Tucson; AZ; USA). (3) To quantify proliferating beta cells, we used double immunofluorescent staining for guinea pig anti-insulin and rabbit anti-Ki67 (1:100; clone SP6; Acris Antibodies GmbH; Herford; Germany) on paraffin sections from both the corpus and tail part of the pancreas. (4) To screen for massive leucocytic infiltrates, every 40^th^ section of five (4 paraffin and 1 frozen) tissue blocks from the tail was stained for mouse anti-CD45 in combination with rabbit anti-synaptophysin (1:5000; Acris). (5) Of two out of three tissues with massive leucocytic infiltrates suspect for TLSs, sufficient sections remained to characterize these structures in detail by using consecutive paraffin- and cryo-sections through staining with guinea pig anti-insulin, rabbit anti-CD3, mouse anti-CD20, rat anti-MECA-79 (1:50; Santa Cruz Biotechnology; Dallas; TX; USA), rabbit anti-CD23 (ready-to-use; clone SP23; Ventana) and rabbit anti-CD21 (ready-to-use; clone EP3093; Ventana) antibodies. (6) One massive leucocytic infiltrate was found in a 50 µm thick cryo-section stained for guinea pig anti-insulin, rabbit anti-CD3 and mouse anti-CD31 (1:50; clone JC/70A; Agilent Technologies).

Pretreatment was carried out with Citrate Buffer (pH 6.0; ScyTek Laboratories; Logan; UT; USA) for anti-Ki67 and anti-synaptophysin or with Tris EDTA Buffer (Klinipath; Olen; Belgium) for anti-CD3, anti-CD68, anti-CD8, anti-CD20, anti-CD4, anti-MECA-79, and anti-CD31.

Binding of primary antibodies was detected with immunofluorescence: DyLight 488, DyLight 549 or Alexa Fluor 647 (1:500; Jackson ImmunoResearch Laboratories; West Grove; PA; USA) or with immunohistochemistry: biotinylated Ig (Vector Laboratories; Burlingame; CA; USA) in combination with Vectastain Elite ABC kit or ABC-AP kit (Vector Laboratories) using Liquid DAB+ Substrate Chromogen System and Fuchsin+ Substrate Chromogen System (Agilent Technologies) as substrate. Sections were mounted with fluorescent mounting medium (Agilent Technologies) containing DAPI (10 µg/ml; Sigma-Aldrich) or with PERTEX mounting medium (Histolab; Göteborg; Sweden).

**Supplementary table**

**Table S1.** Clinical data.

| **Case number DBB3450, Diabetes Biobank Brussels** | | |
| --- | --- | --- |
| **Age (years)** | 66 | |
| **BMI (weight, length)** | 22kg/m² (55kg; 1m58) | |
| **Ethnicity** | Caucasian | |
| **HbA1c** | 13% (118mmol/mol) at diagnosis  8.6% (70mmol/mol) 18 years after diagnosis (2 months before death) | |
| **Plasma C-peptide** | At diagnosis | Fasting: 0.25nmol/l (normal range: 0.20-0.80)  IV glucagon*: 0.41nmol/l (normal range: 0.90-1.90) |
|  | 8 years after diagnosis | Fasting: 0.06nmol/l  IV glucagon*: 0.08nmol/l |
|  | 10 years after diagnosis | 0.19nmol/l at a glycemia of 279mg/dl |
|  | 17 years after diagnosis | 0.05nmol/l at a glycemia of 154mg/dl |
| **Autoantibodies** | GAD: 41U/ml (normal range <1); tested once 6 years after diagnosis (RIA: CentAK anti-GAD65 kit; cut-off value ≥0.9U/ml) | |
|  | ICA: negative; only tested once at time of diagnosis (indirect immunofluorescence assay with human pancreas as substrate; cutoff value ≥4JDF U) | |
| **Comorbidities** | Vitiligo, autoimmune gastritis (positive GPC) and neuropathy (autonomic and peripheral) | |

*Plasma C-peptide concentration in serum 6 minutes after intravenous injection of 1 mg glucagon. GAD: antibodies to glutamic acid decarboxylase, ICA: islet-cell antibodies, GPC: anti-gastric parietal cell antibodies.

**Supplementary figure**


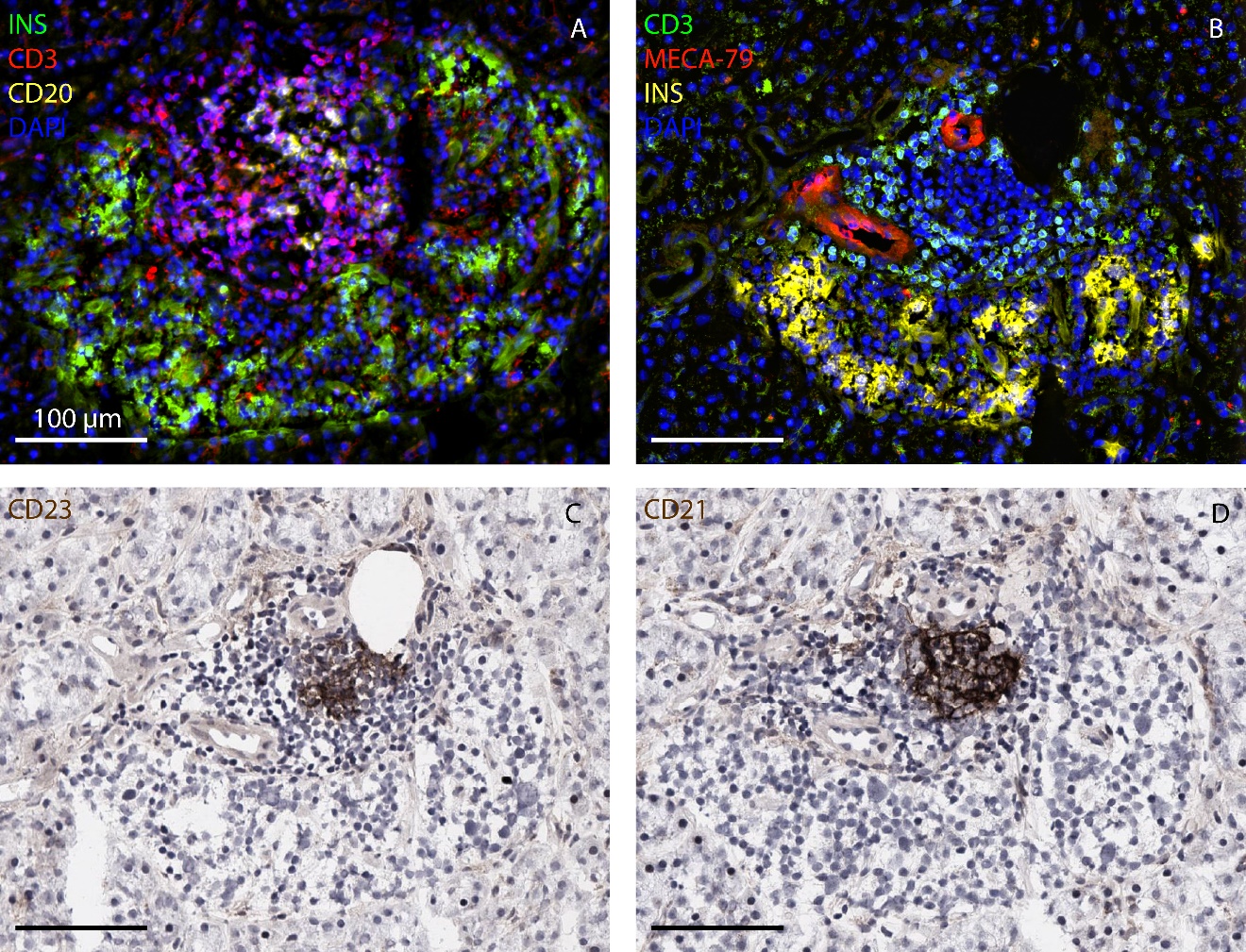


**Figure S1.**

**Legends for supplementary figure**

**Figure S1.** (A) Section stained for insulin (green), CD3 (red) and CD20 (yellow) showing a large lymphocytic infiltrate with a medulla rich in CD20 B-lymphocytes and a peripheral cortex of CD3 T-lymphocytes. (B) Staining of consecutive sections for CD3 (green), MECA-79 (red), insulin (yellow) and DAPI (blue) showing MECA-79 positive high-endothelial venules in the cortex and CD23 (C) or CD21 (D) positive follicular dendritic cells in the medulla, indicating the presence of another TLS-like structure (bar 100 µm).
